# Rapid simultaneous detection of *katG* S315T and *rpoB* S531L mutations for molecular screening of multidrug-resistant tuberculosis using a dual LAMP-lateral flow assay

**DOI:** 10.1128/spectrum.02195-26

**Published:** 2026-10-08

**Authors:** Jutturong Ckumdee, Somchai Santiwatanakul, Nakarin Vutipow, Thongchai Kaewphinit

**Affiliations:** 1 Department of Clinical Pathology, Faculty of Medicine, Vajira Hospital, Navamindradhiraj University 59487, Bangkok, Thailand; 2 Department of Pathology, Faculty of Medicine, Srinakharinwirot University 159248, Nakhonnayok, Thailand; 3 Genetic Engineering and Bioinformatics Interdisciplinary Program, Graduate School, Kasetsart University 504581, Bangkok, Thailand; 4 Center of Excellence in Medical and Environmental Innovation Research (CEMEIR), Srinakharinwirot University 37692, Bangkok, Thailand; 5 Innovative Learning Center, Srinakharinwirot University 37692, Bangkok, Thailand; University of Hawaii at Manoa, Honolulu, Hawaii, USA

**Keywords:** *Mycobacterium tuberculosis*, multidrug-resistant tuberculosis, drug-resistance mutations, loop-mediated isothermal amplification (LAMP), molecular diagnostics

## Abstract

The global spread of multidrug-resistant tuberculosis (MDR-TB) remains a major challenge to tuberculosis control programs, particularly in resource-limited settings where access to rapid molecular drug-susceptibility testing is limited. Resistance to isoniazid and rifampicin is most commonly associated with mutations in the *katG* and *rpoB* genes, respectively, making these loci valuable molecular markers for early MDR-TB screening. In this study, we developed a dual loop-mediated isothermal amplification assay coupled with lateral flow detection for the simultaneous identification of the *katG* S315T and *rpoB* S531L mutations in *Mycobacterium tuberculosis*. Mutation-specific primer sets were designed using mismatch amplification principles to achieve single-nucleotide specificity. Amplification was performed at 61°C for 60 min, followed by visual detection on a three-line lateral flow strip within 5 min. The assay demonstrated a preliminary detection threshold of approximately 10 CFU/mL equivalents and exhibited no cross-reactivity with wild-type *M. tuberculosis* H37Rv, non-tuberculous mycobacteria, or non-target resistance-associated mutations, including the *inhA* −15 promoter mutation and *rpoB* codon 526 variants. Preliminary clinical feasibility was evaluated using genetically characterized clinical sputum specimens and showed complete concordance with GenoType MTBDRplus and Sanger DNA sequencing results. Because the assay targets only two resistance-associated mutations, a negative result does not exclude isoniazid or rifampicin resistance mediated by alternative mutations.

**IMPORTANCE:**

These proof-of-concept findings demonstrate the initial feasibility of a rapid, low-complexity molecular platform for the simultaneous detection of clinically important resistance-associated mutations in *Mycobacterium tuberculosis*. The assay represents a promising step toward low-complexity molecular screening for selected multidrug-resistant tuberculosis-associated mutations, bringing us closer to simplified testing in settings where access to conventional molecular drug-resistance testing remains limited.

## INTRODUCTION

Tuberculosis (TB), caused by *Mycobacterium tuberculosis*, remains one of the leading infectious causes of morbidity and mortality worldwide and continues to pose a major threat to global public health (1, 2). Despite substantial advances in disease control strategies, TB remains responsible for millions of new infections and deaths annually, particularly in low- and middle-income countries (3). According to the World Health Organization (WHO) Global Tuberculosis Report 2024, an estimated 10.8 million individuals developed TB in 2023, resulting in approximately 1.25 million deaths worldwide (4). The global burden of TB is further complicated by the continued emergence and transmission of drug-resistant strains, which threaten progress toward the WHO End TB Strategy and place substantial pressure on healthcare systems (5, 6).

Drug-resistant tuberculosis, particularly multidrug-resistant tuberculosis (MDR-TB), remains a major obstacle to effective TB control (7). MDR-TB is defined as tuberculosis caused by strains resistant to at least isoniazid (INH) and rifampicin (RIF), the two most potent first-line anti-tuberculosis drugs (8). The WHO estimated that approximately 400,000 individuals developed rifampicin-resistant tuberculosis (RR-TB) in 2023, with the majority of these cases meeting the criteria for MDR-TB. Although significant progress has been achieved in the development of novel therapeutic regimens, treatment outcomes for MDR/RR-TB remain substantially poorer than those observed for drug-susceptible TB, largely due to delayed diagnosis, prolonged treatment duration, increased toxicity, and limited access to appropriate therapy in many high-burden regions (9, 10).

The timely detection of drug resistance is essential for improving patient outcomes and limiting the transmission of resistant strains (11). Delays in identifying MDR-TB frequently result in ineffective empirical treatment with first-line regimens, allowing disease progression and prolonged infectiousness before appropriate therapy can be initiated (12). Such delays are associated with increased morbidity, mortality, treatment failure, and healthcare costs (13). Furthermore, undiagnosed or inadequately treated MDR-TB patients continue to serve as reservoirs for community transmission, contributing to the ongoing spread of resistant *M. tuberculosis* lineages (14). Consequently, rapid molecular identification of resistance-associated mutations has become a critical component of modern tuberculosis control programs and antimicrobial resistance (AMR) surveillance initiatives (15).

Conventional phenotypic drug susceptibility testing (DST) remains the reference standard for determining drug resistance in *M. tuberculosis* because it directly measures bacterial growth in the presence of anti-tuberculosis agents (16). However, phenotypic DST requires specialized biosafety laboratories and prolonged incubation periods because *M. tuberculosis* is slow-growing, often delaying results by several weeks (17). To address these limitations, several molecular diagnostic platforms have been developed and recommended by the WHO (18). The Xpert MTB/RIF and Xpert MTB/RIF Ultra assays (Cepheid, Sunnyvale, CA, USA) enable rapid detection of *M. tuberculosis* and rifampicin resistance within a few hours (19), whereas the GenoType MTBDRplus assay (Hain Lifescience GmbH, Nehren, Germany) provides simultaneous identification of mutations associated with resistance to both rifampicin and isoniazid (20). Although these technologies have significantly improved access to rapid drug-resistance testing, their implementation remains constrained in many resource-limited settings due to the need for dedicated instrumentation, specialized laboratory infrastructure, an uninterrupted electricity supply, and relatively high operational costs (21). Consequently, access to rapid molecular drug-susceptibility testing remains uneven across many regions where the burden of tuberculosis is greatest.

Genotypic resistance to INH and RIF is predominantly associated with a limited number of highly conserved mutations within the *M. tuberculosis* genome (22, 23). Among INH-resistant isolates, the *katG* S315T mutation (AGC→ACC) represents one of the most prevalent resistance-associated mutations worldwide and is responsible for a substantial proportion of high-level isoniazid resistance (24, 25). Likewise, more than 95% of rifampicin-resistant strains harbor mutations within the 81-bp rifampicin resistance-determining region (RRDR) of the *rpoB* gene, with the *rpoB* S531L mutation (TCG→TTG) being the most frequently reported variant globally (23, 26). Because these mutations are strongly associated with clinically relevant resistance phenotypes, their rapid identification can provide a practical molecular surrogate for early MDR-TB screening and facilitate timely initiation of appropriate treatment.

Loop-mediated isothermal amplification (LAMP) has emerged as a promising alternative to PCR-based diagnostics because it enables rapid nucleic acid amplification under isothermal conditions using simple heating equipment (27). The method exhibits high analytical sensitivity, rapid amplification kinetics, and tolerance to inhibitory substances, making it particularly attractive for testing in low-complexity laboratory environments (28, 29). Nevertheless, multiplex detection of resistance-associated mutations remains challenging because conventional endpoint detection methods, including turbidity measurements, fluorescent dyes, and agarose gel electrophoresis, are not ideally suited to simultaneously discriminate among multiple targets (30). Integration of LAMP with lateral flow-based detection systems offers an attractive approach for rapid visual interpretation while minimizing instrumentation requirements (31, 32).

Despite recent advances in molecular diagnostics for drug-resistant tuberculosis, there remains a need for simple, rapid, and low-complexity assays capable of simultaneously identifying clinically important resistance-associated mutations. In particular, concurrent detection of the *katG* S315T and *rpoB* S531L mutations could provide a practical strategy that brings us closer to simplified molecular screening of MDR-TB in peripheral or resource-constrained laboratory settings. Therefore, the objective of this study was to develop and evaluate a dual loop-mediated isothermal amplification assay coupled with lateral flow detection (dual LAMP–LFD) for the simultaneous identification of these two clinically significant mutations in *M. tuberculosis*. The analytical performance, mutation specificity, and preliminary clinical feasibility of the assay were subsequently evaluated using genetically characterized clinical sputum specimens and compared with established molecular reference methods.

## MATERIALS AND METHODS

### Clinical specimens and reference characterization

A pre-characterized panel of 39 genetically characterized clinical sputum specimens was included in this study. The specimen panel was intentionally enriched with drug-resistant tuberculosis cases to evaluate the mutation-detection capability of the dual LAMP–LFD assay. Therefore, the distribution of resistance-associated mutations within the study population was not intended to represent the prevalence of drug resistance in the general tuberculosis population. They were obtained from the National Tuberculosis Reference Laboratory, Bureau of Tuberculosis, Department of Disease Control, Ministry of Public Health, Thailand. All specimens had previously undergone routine molecular drug-resistance testing using the GenoType MTBDRplus assay, which served as the primary reference method for detecting resistance-associated mutations in the *katG* and *rpoB* genes.

Among the 39 specimens, 30 carried the *katG* S315T mutation associated with isoniazid resistance, whereas 9 lacked the mutation and were classified as isoniazid-susceptible. Similarly, 28 specimens carried the *rpoB* S531L mutation associated with rifampicin resistance, while 11 were classified as rifampicin-susceptible. These genetically characterized specimens were used to evaluate the diagnostic performance of the dual LAMP–LFD assay. *M. tuberculosis* H37Rv was included as a drug-susceptible wild-type control throughout the study. Nuclease-free water was used as a no-template control (NTC).

### DNA preparation

Genetically characterized clinical sputum specimens were processed using the standard N-acetyl-l-cysteine–sodium hydroxide (NALC–NaOH) decontamination procedure. Following decontamination, specimens were centrifuged, and the resulting pellets were resuspended in sterile distilled water. To ensure laboratory biosafety, suspensions were heat-inactivated at 95°C for 20 min. Samples were subsequently sonicated to facilitate disruption of the mycobacterial cell wall and release of genomic DNA. The resulting lysates were used directly as DNA templates for downstream LAMP analysis.

For exploratory analytical sensitivity studies, bacterial suspensions were prepared in sterile normal saline and adjusted to a turbidity equivalent to a 0.5 McFarland standard (approximately 1.5 × 10^8^ CFU/mL equivalents), followed by serial 10-fold dilutions prior to cell disruption. Two microliters of the resulting DNA preparation were introduced into each reaction. Because DNA concentration and genome-copy number were not quantified after cell disruption, the exact target copy number per reaction was not determined.

### Primer and label design for the isothermal amplification assay

Reference sequences of the *M. tuberculosis katG* gene (GenBank accession no. X68081.1) and *rpoB* gene (GenBank accession no. JX303320) were retrieved from the NCBI database for primer design. LAMP primers were designed using Primer Explorer V5 (Eiken Chemical Co., Ltd., Tokyo, Japan) to recognize eight distinct regions within each target sequence.

Primer design focused on the clinically important *katG* S315T (AGC→ACC) and *rpoB* S531L (TCG→TTG) mutations. To achieve allele-specific amplification, the backward inner primers (BIPs) were engineered with intentional mismatches near the 3′ terminus to selectively amplify mutant alleles while suppressing amplification from wild-type templates. The mismatch optimization strategy used for SNP-specific LAMP was described previously by Ckumdee et al. (33).

For lateral flow detection, the *katG*-specific BIP primer was labeled with biotin, and the *rpoB*-specific BIP primer was labeled with digoxigenin (DIG) at the 5′ terminus. Target-specific FITC-labeled loop probes were incorporated to enable visual detection of amplification products on the lateral flow strip. All oligonucleotides were synthesized and HPLC purified by Bio Basic Inc. (Markham, ON, Canada). Primer and probe sequences are summarized in Table 1.

**TABLE 1:** Primers and probes used for LAMP of the *katG* S315T and *rpoB* S531L gene mutations in isoniazid and rifampicin drug resistance in *M. tuberculosis*

Name	Sequence
F3-*katG*	5′-TTCGGTAAGACCCATGGCG-3′
B3-*katG*	5′-CGAGGAAACTGTTGTCCCAT-3′
FIP-*katG*	5′-AAGCCCATCTGCTCCAGCGG-TTTT-CTGGTCGGCCCCGAACCCG-3′
BIPbio-*katG*	5′-biotin-AGAGCTCGTATGGCACCGGAA-TTTT-CATACGACCTCGATGCAGG-3′
LB FITC-*katG*	5′-FITC-CGGTAAGGACGCGATCACC-3′
F3-*rpoB*	5′-CCGCAGACGTTGATCAACAT-3′
B3-*rpoB*	5′-CCCCTCAGGGGTTTCGA-3′
FIP-*rpoB*	5′-GTGCCGAAGAACTCCTTGATTTTTCGGCCGGTGGTCGCCG-3′
BIP-DIG-*rpoB*	5′-digoxigenin-CAACCCGCTGTCGGGGTTTTTTGCCGGGCCCCAGCGTCA-3′
LB FITC-*rpoB*	5′-FITC-ACCGACAAGCGCCGACTG-3′

### Lateral flow detection platform

**Fig 1:**
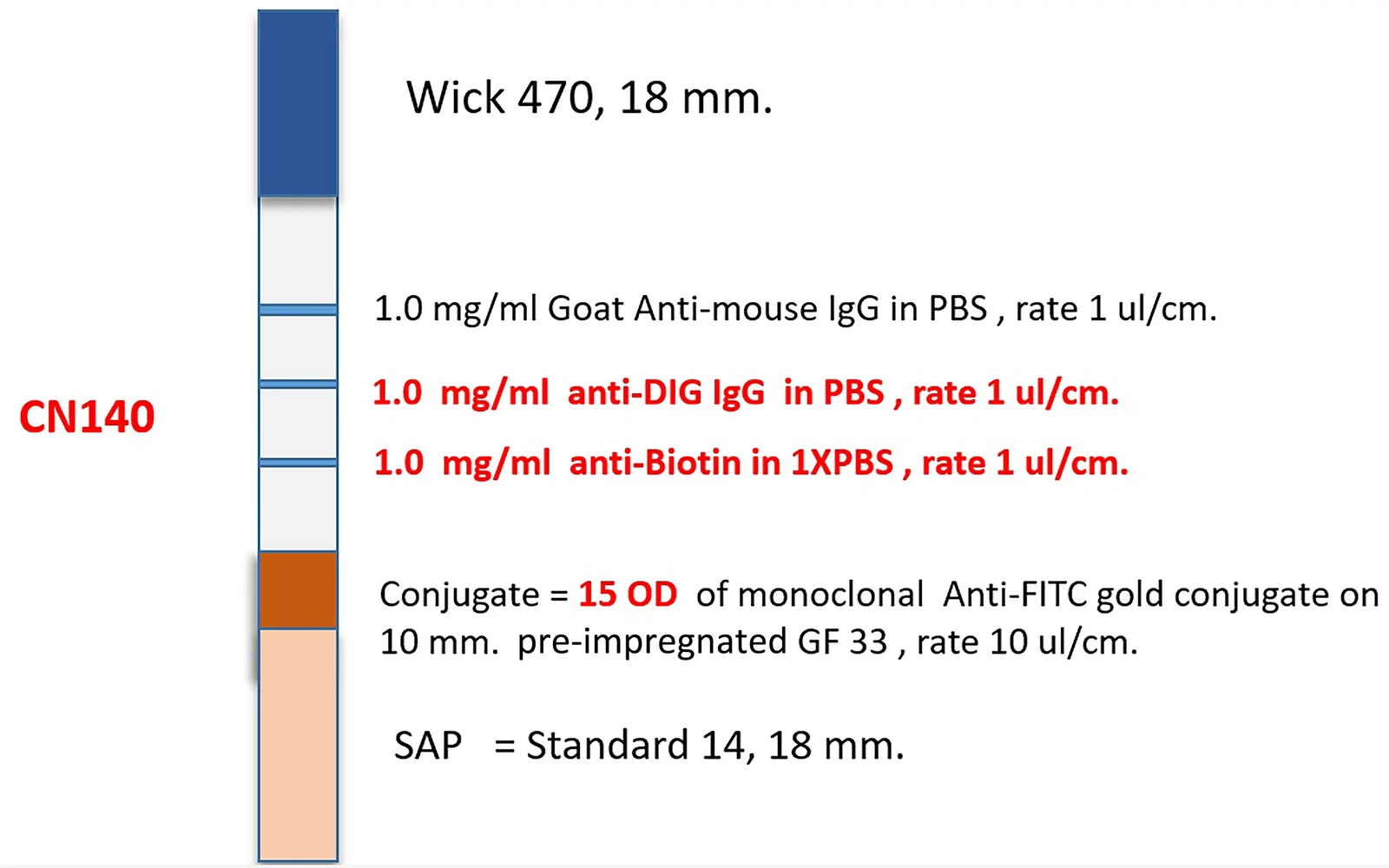
Components and immobilization layout of the three-line lateral flow dipstick (LFD) strip. The diagram depicts the sequential assembly of the sample pad (SAP), gold conjugate pad (15 OD anti-FITC), CN140 nitrocellulose membrane containing anti-biotin IgG (T1), anti-DIG IgG (T2), and goat anti-mouse IgG (C) lines, and the upper wick pad with their respective dimensions and dispensing parameters.

A three-line lateral flow detection (LFD) strip was developed for simultaneous identification of *katG* S315T and *rpoB* S531L amplification products. The strip consisted of a sample pad, a conjugate pad, a nitrocellulose membrane, and an absorbent pad, assembled on an adhesive backing card (Fig. 1).

The first test line (T1) contained anti-biotin antibody for the detection of *katG* amplicons, whereas the second test line (T2) contained anti-DIG antibody for the detection of *rpoB* amplicons. A control line containing goat anti-mouse IgG was included to verify proper chromatographic migration. Anti-FITC gold nanoparticle conjugates were deposited on the conjugate pad to enable visual detection of labeled amplification products (34).

### Dual LAMP–LFD assay

Amplification was performed in a final reaction volume of 25 μL containing 1.6 μM inner primers, 0.2 μM outer primers, 0.2 μM loop primers, 0.2 μM FITC-labeled probes, 1.4 mM dNTPs, 6.0 mM MgSO_4_, 0.5 M betaine, and 8 U of *Bst* 2.0 WarmStart DNA polymerase (New England Biolabs, Ipswich, MA, USA) (35).

Two microliters of DNA template was added to each reaction. Amplification was performed at 61°C for 60 min and terminated at 80°C for 10 min. Amplified products were subsequently analyzed using the LFD strip without any post-amplification hybridization step.

For lateral flow detection, a direct dipstick format was used to minimize liquid handling and aerosol generation. Following amplification, 100 μL of running buffer was added directly into the reaction tube, and the sample pad of the LFD strip was inserted directly into the mixture without transferring amplicons to secondary vessels. Visual results on the strip were interpreted within 5 min, yielding a total post-extraction analytical turnaround time of 75 min. The T1 line indicated detection of the *katG* S315T mutation, whereas the T2 line indicated detection of the *rpoB* S531L mutation. Simultaneous appearance of T1 and T2 indicated the presence of both mutations. The control line alone was interpreted as a negative result. Assays lacking a visible control line were considered invalid and repeated.

To mitigate the risk of amplicon carryover contamination, standard laboratory contamination-control practices and physical partitioning were maintained. Pre-amplification procedures (reagent preparation and template addition) and post-amplification procedures (lateral flow detection and gel electrophoresis) were conducted in strictly segregated laboratory rooms under a unidirectional workflow. Dedicated sets of micropipettes, aerosol-barrier filter tips, and laboratory consumables were assigned to each area. Work surfaces and equipment were routinely decontaminated using 10% sodium hypochlorite followed by 70% ethanol and exposed to ultraviolet (UV) irradiation for at least 30 min before and after each assay run. No-template controls (NTCs) were systematically included in every amplification run to monitor background contamination. An enzymatic carryover-prevention system (such as dUTP/uracil-DNA glycosylase [UDG]) was not incorporated in this study; thus, operational containment remained critical.

### Analytical sensitivity, allele specificity, and preliminary clinical feasibility evaluation

Analytical sensitivity was evaluated in an exploratory dilution series using serial 10-fold dilutions of *M. tuberculosis* ranging from 10^6^ to 10^0^ CFU/mL equivalents performed in triplicate technical replicates (*n* = 3). The lowest concentration yielding visible amplification signals across evaluated replicates was designated as a preliminary detection threshold. A formal analytical limit of detection (LoD), strictly defined as the lowest target concentration producing at least 95% positive reaction rates across extensive replicates, was not formally determined in this proof-of-concept phase and remains to be established.

Analytical allele specificity was assessed using DNA templates carrying the target mutations *katG* S315T and *rpoB* S531L, as well as non-target resistance-associated mutations, including *inhA* −15 C→T and *rpoB* codon 526 variants. Wild-type *M. tuberculosis* H37Rv and representative non-tuberculous mycobacteria (*M. intracellulare*, *M. fortuitum*, and *M. avium*) were included as specificity controls.

Clinical applicability was evaluated using the 39 characterized sputum specimens. Results obtained by dual LAMP–LFD were compared with those generated by GenoType MTBDRplus, phenotypic drug susceptibility testing (standard proportion method on Löwenstein–Jensen medium), and Sanger DNA sequencing. For sequence confirmation, PCR amplicons encompassing the *katG* codon 315 and *rpoB* codon 531 regions were subjected to bidirectional sequencing. Sequence data were aligned with reference *M. tuberculosis* sequences to verify the presence or absence of resistance-associated mutations. Sanger sequencing results were used as the definitive molecular reference standard. Because complete agreement was observed between the dual LAMP–LFD assay and the reference methods, the preliminary mutation detection concordance was summarized descriptively. Exact two-sided 95% confidence intervals (CIs) for sensitivity, specificity, and overall concordance were calculated using the Clopper-Pearson method. Because of the small, preselected, resistance-enriched specimen panel, these measures were interpreted as descriptive estimates of preliminary mutation-detection concordance rather than definitive estimates of clinical diagnostic accuracy.

## RESULTS

### Optimization of amplification temperature for the dual LAMP–LFD assay

To establish the optimal amplification temperature for the dual LAMP–LFD assay, reactions were performed using the previously developed mutant-specific primer sets targeting the *katG* S315T (AGC→ACC) and *rpoB* S531L (TCG→TTG) mutations. Amplification was carried out at 61°C, 63°C, 65°C, and 68°C under otherwise identical reaction conditions. Following incubation, the amplification products were analyzed by both agarose gel electrophoresis and LFD detection.

**Fig 2:**
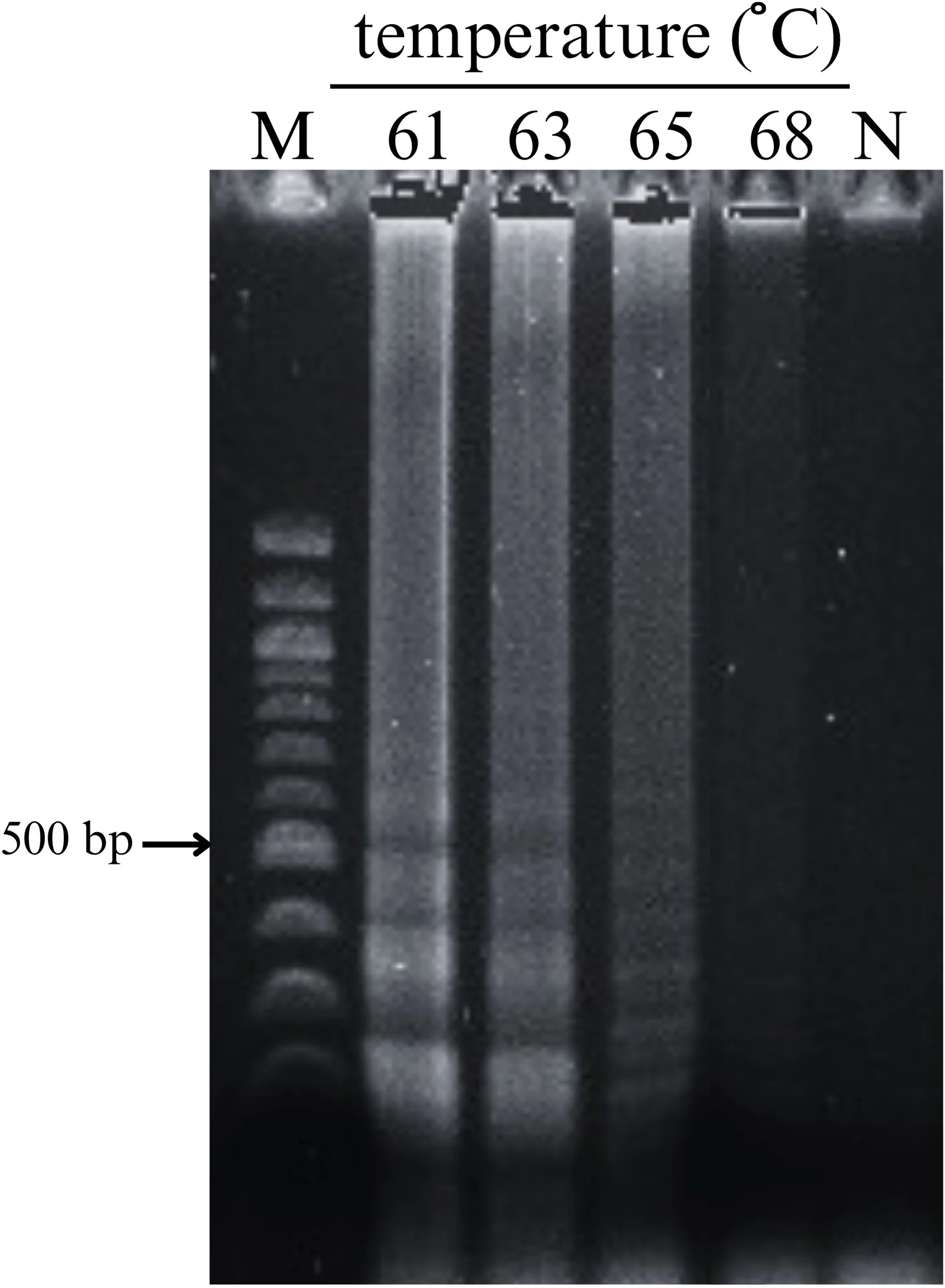
Effect of incubation temperature on the amplification efficiency of the dual LAMP assay. Gel lanes represent a DNA marker with a 500-bp reference band (Lane M), assay optimization at different temperature points (lanes 61°C, 63°C, 65°C, and 68°C), and the negative control (lane N).

Agarose gel electrophoresis revealed the characteristic ladder-like banding pattern of LAMP amplification at all tested temperatures, indicating successful amplification of both target loci (Fig. 2). Although positive amplification was observed across the entire temperature range, differences in amplification efficiency were evident from the relative intensity of the ladder-like amplicons. The strongest and most distinct amplification profiles were observed at 61°C, whereas slightly reduced product intensities were observed at 63–68°C.

### Analytical sensitivity of the dual LAMP–LFD assay

The analytical sensitivity of the dual LAMP–LFD assay was evaluated using ten-fold serial dilutions of *M. tuberculosis* genomic DNA corresponding to bacterial concentrations ranging from 10^6^ to 10^0^ CFU/mL equivalents.

**Fig 3:**
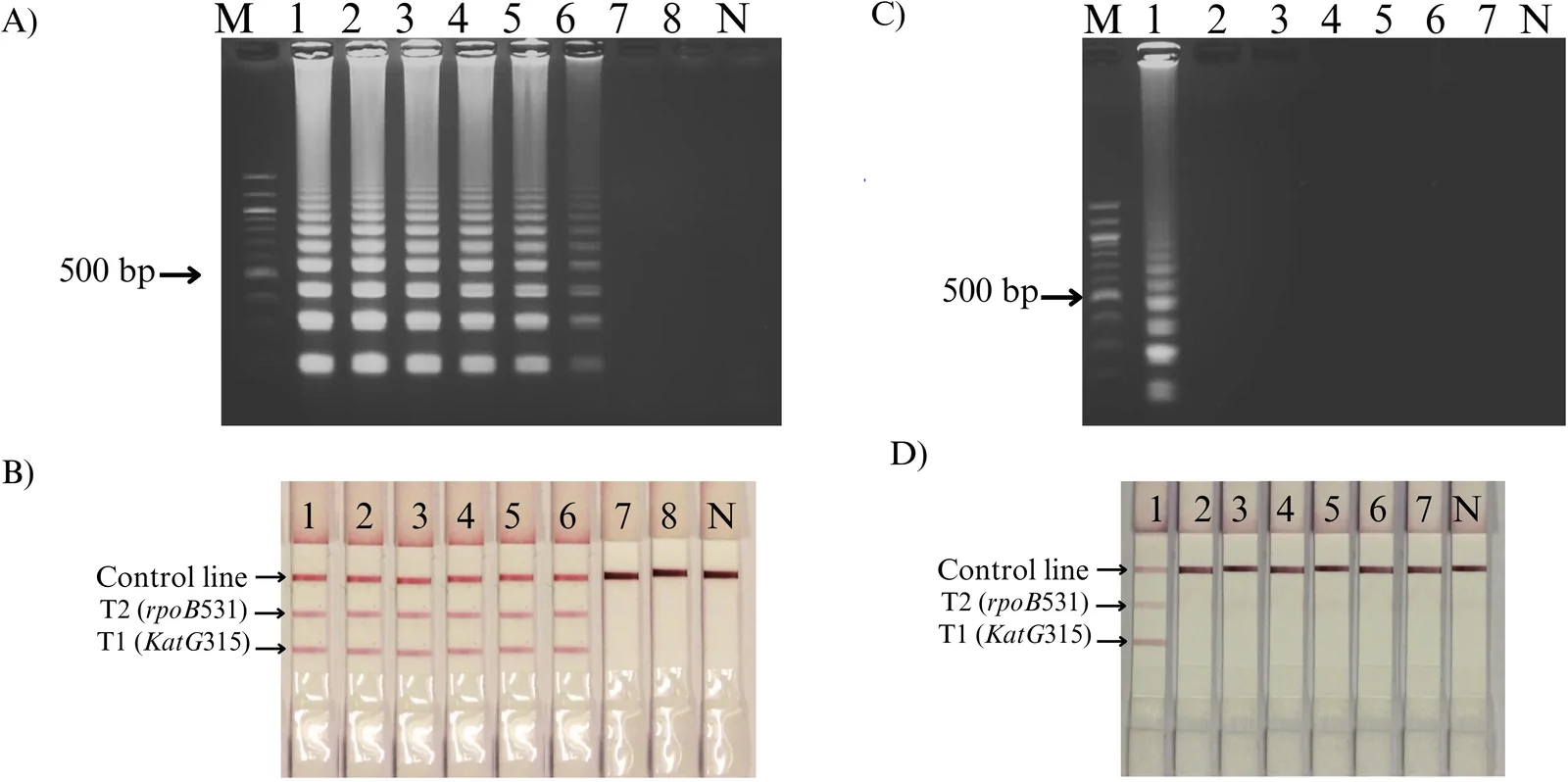
Analytical sensitivity and specificity of the dual LAMP–LFD assay. (**A**) 2% agarose gel electrophoresis showing characteristic ladder-like LAMP amplicons. Lane M: DNA marker (arrow indicates the 500 bp band); lanes 1–6: amplification profiles corresponding to bacterial concentrations from 10^6^ to 10^1^ CFU/mL equivalents, respectively; lane 7: 10^0^ CFU/mL equivalents; lane 8: wild-type *M. tuberculosis* H37Rv template; lane N: no-template control. (**B**) Corresponding three-line lateral flow dipstick (LFD) strips confirming successful visual detection of both *katG* S315T (T1) and *rpoB* S531L (T2) lines down to a preliminary detection threshold of 10^1^ CFU/mL equivalents (lane 6). (**C**) Analytical specificity testing of the dual LAMP–LFD assay against non-target mutations and NTM evaluated by 2% agarose gel electrophoresis. Lane M: DNA marker (arrow indicates the 500 bp band); lane 1: *M. tuberculosis* harboring dual *rpoB* S531L and *katG* S315T mutations; lane 2: wild-type *M. tuberculosis* H37Rv; lane 3: *rpoB* codon 526 mutant; lane 4: *inhA* −15 promoter mutant; lane 5: *M. intracellulare*; ane 6: *M. fortuitum*; lane 7: *M. avium*; and lane N: no-template control. Only the targeted dual-mutant template generated positive T1 and T2 visual lines. (**D**) Corresponding lateral flow dipstick strips showing concordance specificity results to agarose gel electrophoresis.

Agarose gel electrophoresis demonstrated characteristic ladder-like amplification products at concentrations ranging from 10^6^ to 10^1^ CFU/mL equivalents (Fig. 3A). Amplification intensity progressively decreased as template concentration declined, whereas no detectable amplification was observed at lower concentrations or in the negative controls.

Consistent with the gel electrophoresis results, lateral flow detection generated visible test-line signals corresponding to both *katG* S315T (T1) and *rpoB* S531L (T2) mutations across the same concentration range (Fig. 3B). Positive signals remained detectable at 10^1^ CFU/mL equivalents, whereas only the control line was observed in reactions containing lower template concentrations, wild-type *M. tuberculosis* H37Rv DNA, or no-template controls.

Under the conditions evaluated in this exploratory dilution experiment, 10^1^ CFU/mL equivalents was the lowest tested concentration producing a visible signal.

### Analytical allele specificity of the dual LAMP–LFD assay

The ability of the dual LAMP–LFD assay to discriminate clinically relevant drug-resistance mutations from non-target mutations was evaluated using a panel of characterized *M. tuberculosis* strains and non-tuberculous mycobacteria.

Positive amplification was observed only in templates carrying the target *katG* S315T and *rpoB* S531L mutations (Fig. 3C). No amplification products were detected in wild-type *M. tuberculosis* H37Rv, strains carrying non-target resistance-associated mutations including *inhA* −15 C→T and *rpoB* codon 526 variants, or representative non-tuberculous mycobacteria (*M. intracellulare*, *M. fortuitum*, and *M. avium*).

Similarly, lateral flow analysis produced positive signals only when the corresponding target mutations were present (Fig. 3D). No cross-reactivity was observed with wild-type strains, non-target resistance mutations, or non-tuberculous mycobacteria. These findings demonstrate the analytical allele specificity of the assay in selectively detecting the *katG* S315T and *rpoB* S531L mutations without cross-reactivity across the panel of wild-type, non-target mutations, and NTM species evaluated in this study.

### Mutation discrimination and genotype verification

**Fig 4:**
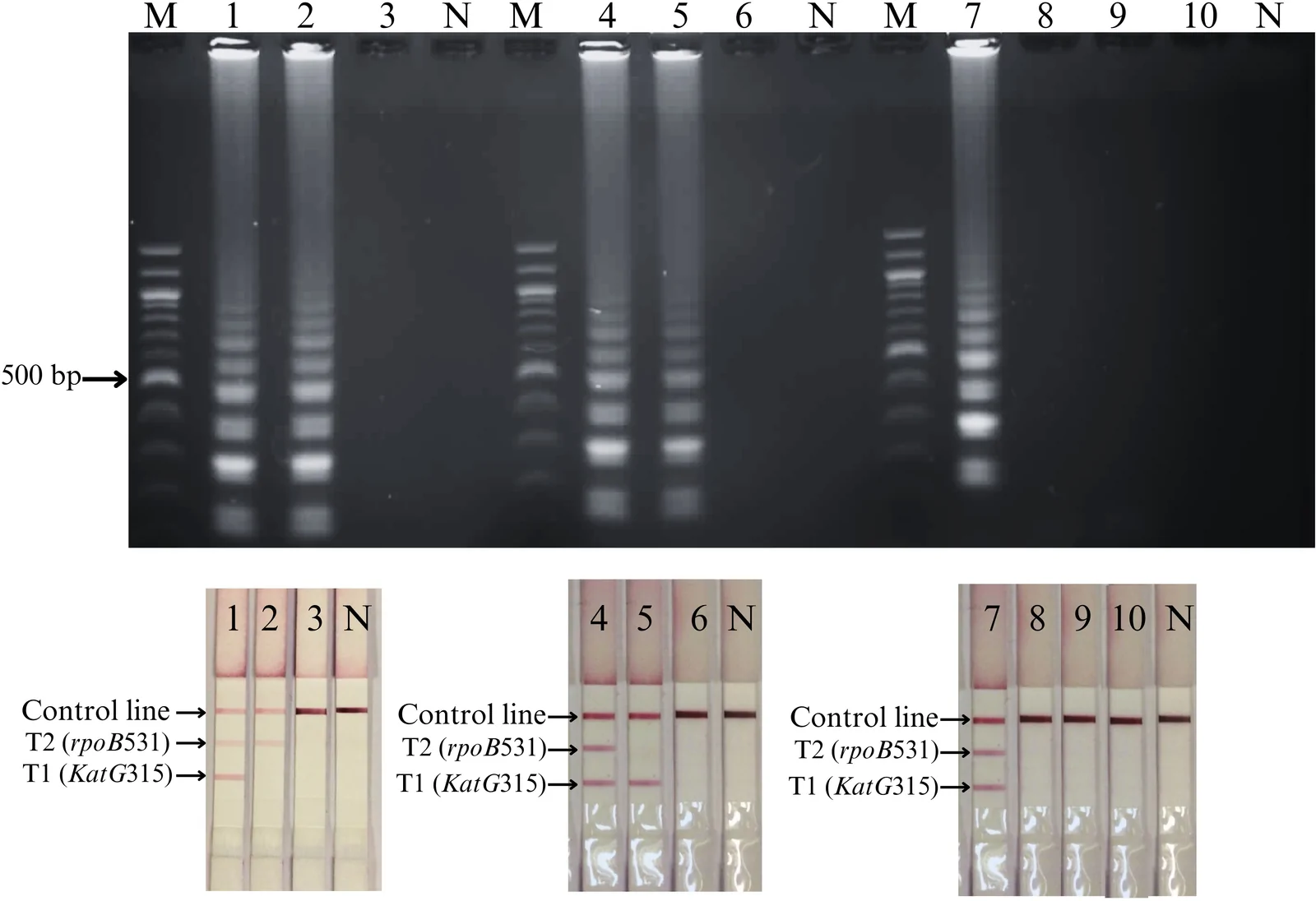
Analytical allele specificity of the dual LAMP–LFD assay evaluated by 2% agarose gel electrophoresis (top) and corresponding three-line lateral flow dipsticks (bottom). Lane M: DNA marker (arrow indicates the 500 bp band); lanes 1 and 7: *M. tuberculosis* clinical isolates harboring both *katG* S315T and *rpoB* S531L mutations (MDR-TB profile showing T1, T2, and control lines); lane 2: *M. tuberculosis* with *rpoB* S531L mutation only (showing T2 and control lines); lane 5: *M. tuberculosis* with *katG* S315T mutation only (showing T1 and control lines); lanes 3, 6, and 8: wild-type *M. tuberculosis* H37Rv templates; lane 9: non-target *rpoB* codon 526 mutant control; lane 10: non-target *inhA* −15 promoter mutant control; lanes N: no-template control. Wild-type, non-target mutations, and no-template control templates displayed only the control line, confirming analytical allele-specificity across the evaluated panel.

To further evaluate mutation-specific discrimination, the dual LAMP–LFD assay was tested using *M. tuberculosis* templates representing different resistance-associated genotypes (Fig. 4).

Samples carrying both *katG* S315T and *rpoB* S531L mutations showed positive signals at both T1 and T2, consistent with a multidrug-resistant tuberculosis genotype. Samples containing only the *katG* S315T mutation generated a positive T1 signal, whereas samples containing only the *rpoB* S531L mutation generated a positive T2 signal. In contrast, wild-type strains and isolates carrying non-target mutations yielded only the control line.

These findings indicate that the assay accurately distinguished target mutations from closely related non-target resistance-associated mutations and enabled the simultaneous identification of clinically relevant multidrug-resistant tuberculosis markers in a single reaction.

To verify the genetic identity of the evaluated strains, Sanger DNA sequencing was performed as the molecular reference method. Representative chromatograms confirmed the presence of the AGC→ACC substitution at codon 315 of *katG* and the TCG→TTG substitution at codon 531 of *rpoB*. Complete agreement was observed between sequencing results and dual LAMP–LFD outcomes, supporting the assay’s mutation-specific accuracy.

### Evaluation using genetically characterized clinical sputum specimens

The characteristics of the genetically characterized clinical sputum specimens included in this study are summarized in Table 2. The specimen panel was intentionally enriched with drug-resistant tuberculosis cases to facilitate evaluation of mutation-specific detection performance.

**TABLE 2:** Characteristics of genetically characterized clinical sputum specimens included in the study

Characteristic	Number of specimens (*n* = 39)
Total sputum specimens	39
*katG* S315T-positive	30
*katG* S315T-negative	9
*rpoB* S531L-positive	28
*rpoB* S531L-negative	11
Dual mutation (*katG* S315T + *rpoB* S531L)	28
*katG* S315T only	2
*rpoB* S531L only	0
Neither mutation detected	9

The clinical applicability of the dual LAMP–LFD assay was evaluated using 39 pre-characterized sputum specimens. The distribution of mutation profiles is summarized in Table 2. Within this clinical panel, 28 specimens harbored concurrent dual mutations, 2 specimens contained only the *katG* S315T mutation, 9 specimens lacked either target mutation, and no specimens in this archived cohort harbored an isolated *rpoB* S531L mutation alone. Therefore, while single-target detection of *rpoB* S531L was confirmed during analytical evaluations, clinical feasibility for *rpoB* S531L was evaluated in the context of dual-mutant sputum specimens. Complete agreement was observed between dual LAMP–LFD, GenoType MTBDRplus, phenotypic drug susceptibility testing, and Sanger DNA sequencing for detection of both *katG* S315T and *rpoB* S531L mutations (Table 3). No discordant results were identified. Within this pre-characterized panel, the preliminary sensitivity was 100.0% (95% CI: 88.4%–100.0%) for *katG* S315T and 100.0% (95% CI: 87.7%–100.0%) for *rpoB* S531L. Preliminary specificity was 100.0% (95% CI: 66.4%–100.0%) for *katG* S315T (9/9) and 100.0% (95% CI: 71.5%–100.0%) for *rpoB* S531L (11/11). These statistical indices are explicitly framed as descriptive metrics within this preselected, resistance-enriched evaluation panel rather than definitive indicators of real-world clinical diagnostic accuracy. The relatively wide confidence intervals, particularly for specificity, reflect the small control sample sizes in this preliminary feasibility evaluation.

**TABLE 3:** Concordance of the dual LAMP–LFD assay with reference methods for detection of resistance-associated mutations in genetically characterized clinical sputum specimens

Target mutation	Reference-positive specimens	Reference-negative specimens	LAMP–LFD positive	LAMP–LFD negative	%Sensitivity (95% CI)	%Specificity (95% CI)	%Concordance (95% CI)
*katG* S315T	30	9	30	9	100 (88.4–100.0)	100 (66.4–100.0)	100 (91.0–100.0)
*rpoB* S531L	28	11	28	11	100 (87.7–100.0)	100 (71.5–100.0)	100 (91.0–100.0)

## DISCUSSION

The present study employed a pre-characterized, resistance-enriched clinical specimen panel to assess mutation-specific detection performance. Accordingly, the distribution of *katG* S315T and *rpoB* S531L mutations observed in this study should be interpreted solely within the context of assay evaluation and not as an estimate of mutation prevalence among tuberculosis patients.

The emergence and spread of MDR-TB, primarily driven by genetic mutations conferring resistance to INH and RIF, pose a substantial challenge to global tuberculosis control. Rapid genotypic drug susceptibility testing with minimal instrumentation requirements is essential to bypass lengthy culture-based methods and break the chain of transmission. In this study, we successfully developed and conducted a proof-of-concept evaluation of a dual loop-mediated isothermal amplification assay coupled with a three-line lateral flow dipstick (dual LAMP–LFD) for targeted molecular screening of selected MDR-TB-associated mutations, specifically focusing on the two most clinically prevalent resistance mutations: *katG* S315T and *rpoB* S531L.

Rapid identification of drug-resistant tuberculosis is essential for the timely initiation of appropriate therapy and the interruption of community transmission. Because the *katG* S315T and *rpoB* S531L mutations collectively account for a substantial proportion of global isoniazid- and rifampicin-resistant tuberculosis cases, simultaneous detection of these mutations may provide a practical molecular screening strategy for identifying patients at high risk of MDR-TB. The simplicity of the assay, together with its minimal instrumentation requirements, represents a practical step toward molecular screening in intermediate-tier and resource-constrained laboratories where access to automated molecular platforms remains limited.

A primary advantage of this platform is the integration of mismatch-targeted SNP-LAMP primers with a multi-line immunochromatographic strip, enabling a direct, hybridization-free visual readout. Conventional line-probe assays and multiplex hybridization protocols typically require extensive post-amplification incubations, multiple washing steps, and repeated amplicon transfers, which increase technical complexity and assay time. In contrast, our assay allows direct immersion of the LFD strip straight into the reaction tube following the addition of running buffer, avoiding liquid transfer between vessels and reducing open-tube manipulation. This direct dipstick approach provides a simple, low-complexity alternative to multi-step hybridization assays.

The analytical performance evaluations underscored the analytical sensitivity and target discrimination capability of the dual LAMP–LFD assay against the panel evaluated in this study. The preliminary detection threshold was approximately 10^1^ CFU/mL equivalents under the exploratory conditions evaluated. However, a formal analytical LoD based on ≥95% detection probability across an appropriately designed series of independent replicate experiments remains to be established. While direct head-to-head comparative analytical limit-of-detection studies against automated platforms or multiplex PCR assays were not performed, the observed preliminary detection threshold highlights the potential of this isothermal strategy for detecting low bacterial target levels in basic laboratory settings. This analytical detection capability is fundamentally attributed to the deployment of six primers recognizing eight distinct regions per target gene, driving exponential DNA production under optimal isothermal conditions (61°C).

Furthermore, our assay demonstrated allele-specific detection within the evaluated panel. A common pitfall for isothermal diagnostic platforms is false-positive signaling triggered by nonspecific extension from closely related wild-type configurations or alternative non-target resistance mutations located within the same hypervariable regions (such as the RRDR domain of *rpoB*). To counter this constraint, the 3′ termini of the B2 regions in the BIP architecture were strategically engineered with localized nucleotide mismatches. This design strongly inhibits nonspecific polymerase extension when hybridized to non-mutant templates. As supported by both our LFD phenotypic readouts and gold-standard Sanger DNA sequencing, the system generated positive signals only at T1 and T2 in the presence of the exact *katG* S315T and *rpoB* S531L variants. Crucially, no cross-reactivity or false-positive bands were detected against wild-type *M. tuberculosis* H37Rv, NTM species (*M. intracellulare*, *M. fortuitum*, and *M. avium*), or other major confounding mutations, including the *inhA* −15 promoter and *rpoB* codon 526 variants.

The diagnostic feasibility of the developed dual LAMP–LFD assay was further supported by evaluating 39 clinical *M. tuberculosis* specimens. Complete concordance was observed among our visual LFD outputs, the GenoType MTBDRplus assay, and Sanger sequencing within this preselected panel, supporting the feasibility of the dual LAMP–LFD framework as an initial screening tool for selected resistance-associated mutations. Given that the entire workflow—including amplification, enzyme inactivation, and lateral flow readout—can be completed within approximately 75 min using a standard water bath or heat block, this system provides a low-complexity diagnostic framework that works toward simplified testing workflows in resource-constrained clinical settings.

Despite the promising outcomes and analytical feasibility demonstrated by this platform, several limitations must be addressed to guide future clinical optimization. Primary among these is that the platform’s feasibility was evaluated using a relatively small, pre-characterized panel of clinical sputum specimens (*n* = 39) enriched for drug-resistant tuberculosis. Although this enriched panel facilitated rigorous evaluation of mutation-specific detection capability, the observed mutation frequencies may not reflect those encountered in routine clinical practice. Moreover, within this archived clinical panel, specimens harboring an isolated *rpoB* S531L mutation alone were not represented, meaning that clinical feasibility for this locus was demonstrated primarily within dual-resistant backgrounds. In addition, because anonymized archival specimens were used, baseline clinical characteristics such as quantitative smear microscopy grades (AFB smear status) and exact bacillary loads were not available for stratified analysis. Diagnostic sensitivity and detection thresholds may vary in paucibacillary or smear-negative clinical specimens, which warrants dedicated prospective clinical investigations across stratified bacillary strata. Therefore, larger multicenter prospective diagnostic trials involving unselected patient populations will be required to rigorously compute definitive diagnostic sensitivity, specificity, and predictive values. Additionally, while *katG* S315T and *rpoB* S531L account for the vast majority of first-line drug-resistance global lineages, alternative mutations (such as alternative *rpoB* codons and the *inhA* promoter region) are not covered by the current primer design. Critically, a negative dual LAMP–LFD result does not imply drug susceptibility, as resistance mediated by alternative genetic loci or non-target mutations remains undetected by this assay. Therefore, this platform serves strictly as an initial rule-in screening tool for selected high-frequency mutations, and negative specimens still require standard phenotypic or broad-range molecular drug susceptibility testing when clinical suspicion remains. Future multiplex designs incorporating additional *katG*, *inhA*, and *rpoB* mutations may further expand diagnostic coverage across diverse MDR-TB populations. Furthermore, the long-term chemical stability and shelf life of the fabricated three-line LFD strips and the multi-component isothermal reaction mixture have not yet been systematically characterized. Future investigations must prioritize real-time, accelerated stability monitoring across varied ambient conditions, alongside the deployment of chemical stabilizers or lyophilization protocols, to ensure resilient field storage and shelf-life viability during practical deployment.

Another important limitation of the present workflow is its reliance on standard NALC–NaOH chemical decontamination, centrifugation, and sonication for sample preparation. While the downstream dual LAMP–LFD reaction requires only basic heating equipment, the upstream extraction procedures still require basic biosafety facilities and laboratory infrastructure. Therefore, true decentralized or point-of-care implementation remains aspirational until this isothermal platform is coupled with an instrument-free, rapid sputum lysis or direct extraction-free protocol. Future investigations will evaluate assay performance using unprocessed sputum specimens and simplified sample preparation methods.

Although our direct-immersion dipstick format minimizes pipetting and avoids amplicon transfers between secondary vessels, opening reaction tubes containing high-copy-number LAMP products still poses an inherent risk of aerosol-mediated carryover contamination, particularly in routine or decentralized settings. While this risk was managed in our study through strict physical partitioning, unidirectional workflow, and pre/post-amplification isolation, an enzymatic carryover-prevention system (e.g., heat-labile UDG/dUTP incorporation) was not implemented. Future refinements will explore enzymatic decontamination strategies or fully enclosed, self-contained reaction cassettes that enable visual readout without ever breaching the amplicon tube seal.

A descriptive comparison between the proposed dual LAMP–LFD assay and established diagnostic methods for drug-resistant tuberculosis is summarized in Table 4. Notably, the reported ~75-min turnaround time for the dual LAMP–LFD assay represents post-extraction analytical time (amplification and visual lateral flow detection) and does not include the upstream manual sputum decontamination, centrifugation, heat inactivation, and sonication steps, whereas platforms such as Xpert MTB/RIF Ultra reflect automated, sample-to-result workflows. This comparison is intended solely as an informative methodological overview and does not imply diagnostic equivalence or superiority over thoroughly validated commercial platforms.

**TABLE 4:** Comparison of the dual LAMP–LFD assay with currently available molecular diagnostic methods for multidrug-resistant tuberculosis

Method	Target gene/locus coverage	Upstream sample preparation
Phenotypic DST	Growth phenotype across anti-TB drug panels	Decontamination and culture isolation required
GenoType MTBDRplus	*katG*, *inhA* promoter, and *rpoB* RRDR	Manual extraction (chemical lysis/spin)
Xpert MTB/RIF Ultra	*rpoB* RRDR, *IS*6110, and *IS*1081	Direct sputum buffer addition (minimal manual handling)
Dual LAMP–LFD (this study)	*katG* S315T and *rpoB* S531L variants	Standard NALC–NaOH, centrifugation, and sonication

Method	Turnaround time (TAT)	Degree of automation
Phenotypic DST	Weeks to months	Manual/semi-automated
GenoType MTBDRplus	~1 working day (post-extraction)	Manual (amplification, hybridization, and wash)
Xpert MTB/RIF Ultra	~2 h (true sample-to-result)	Fully automated (hands-off cartridge integration)
Dual LAMP–LFD (this study)	~75 min (post-extraction)	Manual (isothermal incubation and direct dipstick readout)

Method	Validation status	
Phenotypic DST	Established global reference standard	
GenoType MTBDRplus	Widely validated, WHO-endorsed	
Xpert MTB/RIF Ultra	Extensively validated, WHO-endorsed	
Dual LAMP–LFD (this study)	Preliminary proof-of-concept/exploratory clinical feasibility	

### Conclusion

In conclusion, this study provides an analytical proof-of-concept and preliminary clinical feasibility evaluation of a dual LAMP–LFD assay for the simultaneous detection of the *katG* S315T and *rpoB* S531L mutations in *M. tuberculosis*. The assay demonstrated mutation-specific detection within the analytical and preselected clinical panels evaluated. However, negative results do not exclude resistance mediated by alternative mutations, and the current workflow requires laboratory-based sputum processing. Larger prospective studies, expanded variant panels, formal determination of the analytical limit of detection, and simplified sample preparation will be required before clinical or decentralized implementation.

## Data Availability

All data generated or analyzed during this study are included in this published article. Additional data are available from the corresponding author upon reasonable request.
